# Supplementary material for: Antidromic-rectifying gap junctions amplify chemical transmission at functionally mixed electrical-chemical synapses
Source: Nat Commun. 2017 Mar 20;8:14818. doi: 10.1038/ncomms14818 (PMC5364397; doi:10.1038/ncomms14818)
Supplement: Supplementary Information — Supplementary Figures. [file ncomms14818-s1.pdf]

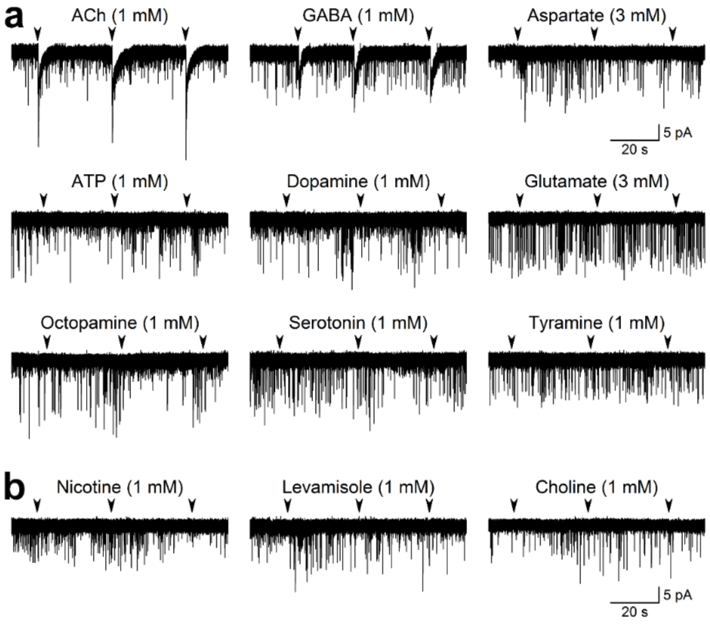

Supplementary Figure 1. Responses of VA5 to exogenous neurotransmitters and agonists. (**a**) Acetylcholine (ACh) and γ-aminobutyric acid (GABA) but not other exogenous neurotransmitters cause inward current. (**b**) Nicotine, levamisole and choline do not cause a current.

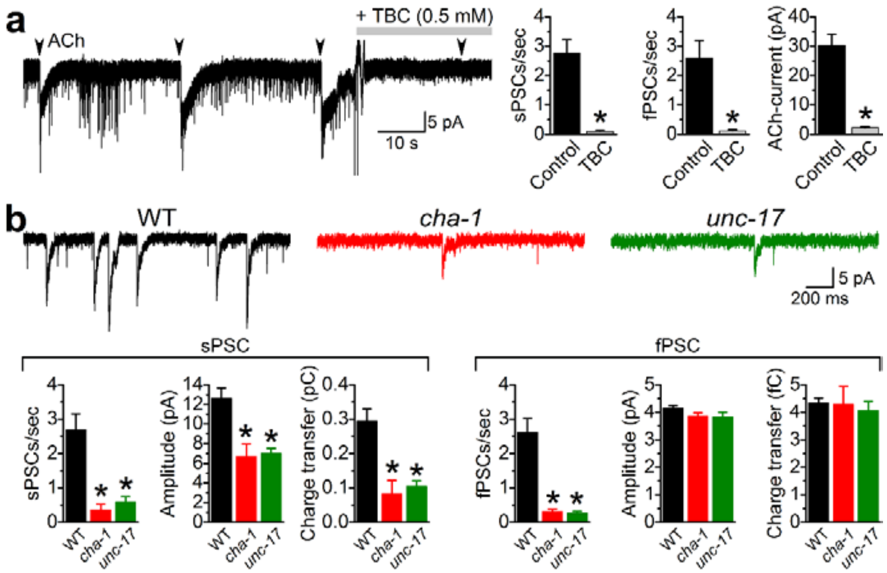

Supplementary Figure 2. Postsynaptic currents (PSCs) in VA5 result from acetylcholine release. (**a**) d-Tubocurarine (TBC) abolishes both exogenous acetylcholine (ACh, 1 mM)-induced whole-cell current and spontaneous PSCs in VA5. The frequencies of slow and fast PSCs (sPSCs and fPSCs) and the amplitude of ACh-induced current are compared between the control period and TBC treatment period. Sample size (n) was 6. (**b**) Comparison of VA5 sPSCs and fPSCs among wild type (WT) (n = 11), *cha-1(p1152)* (n = 5), and *unc-17(e245)* (n = 6). The asterisk (*) indicates a statistically significant difference ($p < 0.05$) based on either paired *t*-test (**a**) or one-way *ANOVA* with Turkey's post hoc test (**b**).

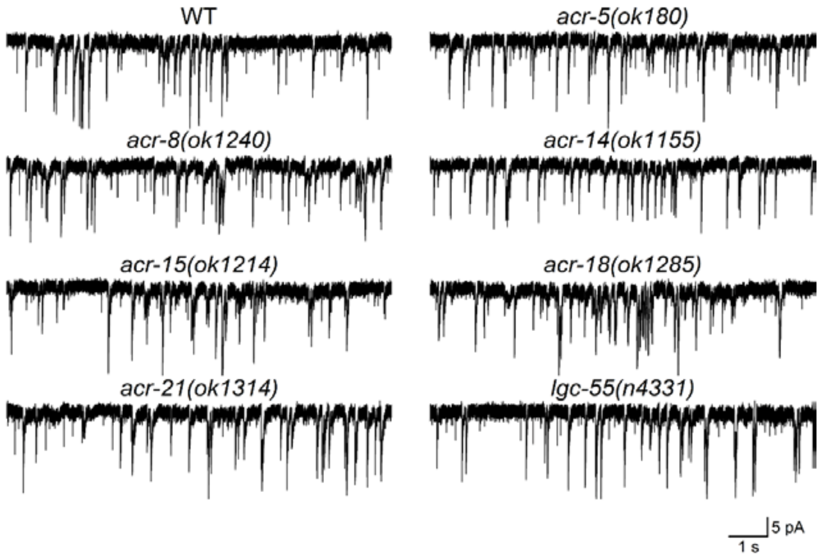

Supplementary Figure 3. Slow postsynaptic currents (sPSCs) appear to be normal in mutants of other tested receptor genes. Shown are sample traces of 2-3 experiments with each mutant strain.

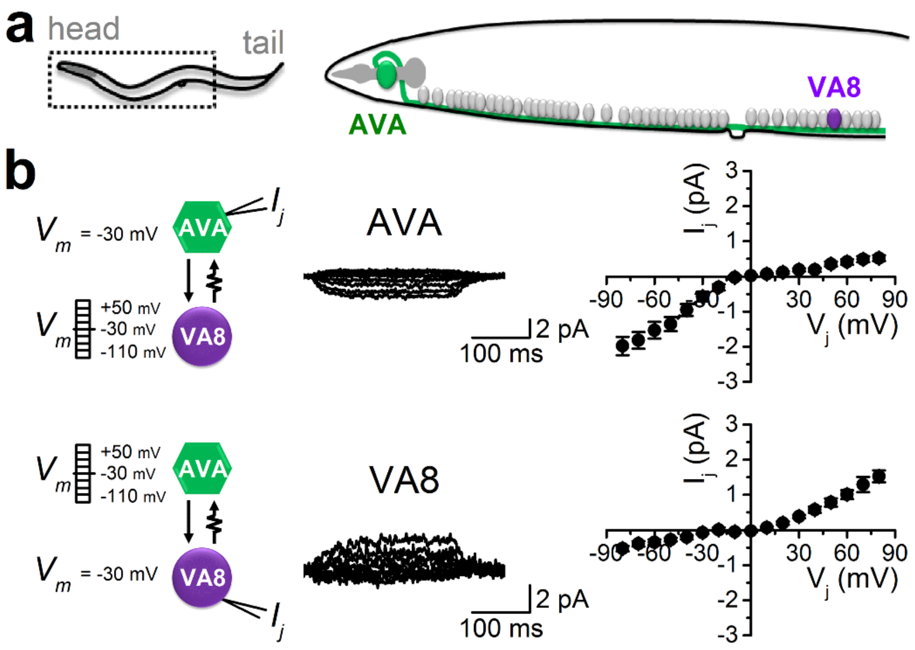

Supplementary Figure 4. Gap junctions between AVA and VA8 mediate antidromic current. (**a**) Diagram showing the locations of AVA and VA8. (**b**) I_j between AVA and VA5 in wild type is strongly rectifying in the AVA direction ($n = 7$). I_j was recorded from one neuron held constantly at −30 mV (neuron #1) while the other neuron (neuron #2) was stepped to various membrane voltages (V_m) from a holding voltage of −30 mV. $V_j = V_m$ of neuron #1 − V_m of neuron #2. Whole-cell membrane currents of the neuron receiving the voltage steps are not shown for clarity.

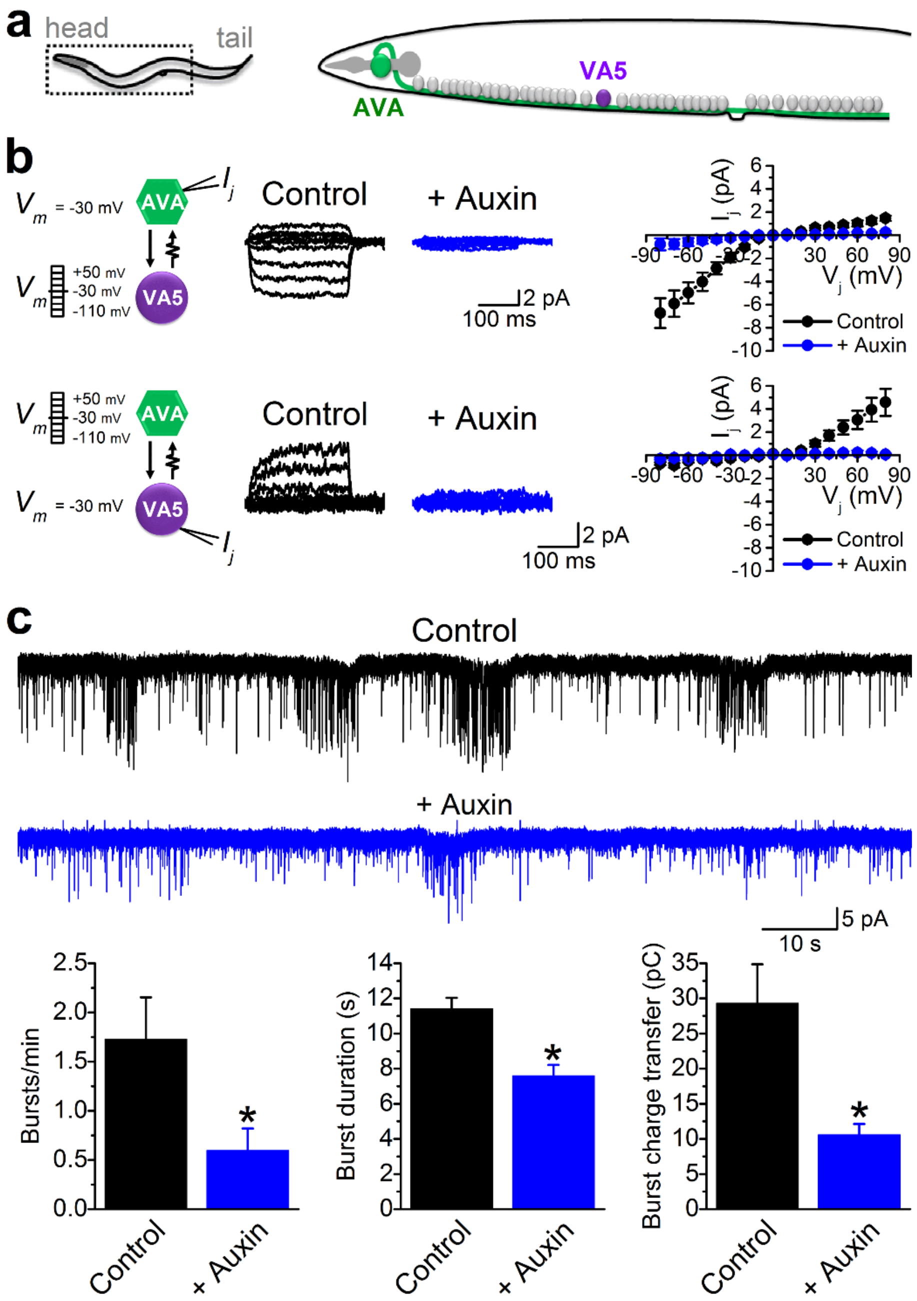

Supplementary Figure 5. Auxin-induced degradation of UNC-7 in AVA inhibits chemical synaptic transmission between AVA and VA5. (**a**) Diagram showing the locations of AVA and VA5. (**b**) In a transgenic strain expressing degron-tagged UNC-7 specifically in AVA, I_j is abolished in worms with auxin pretreatment (+ Auxin, n = 7) but not in those without auxin pretreatment (Control, n = 5). I_j was recorded from one neuron held constantly at −30 mV (neuron #1) while the other neuron (neuron #2) was stepped to various membrane voltages (V_m) from a holding voltage of −30 mV. $V_j = V_m$ of neuron #1 − V_m of neuron #2. Whole-cell membrane currents of the neuron receiving the voltage steps are not shown for clarity. (**c**) UNC-7 degradation in AVA induced by auxin pretreatment (n = 7) causes great inhibition of PSC bursts in VA5 compared with the control (no auxin, n = 8). Data are shown as mean ± SE. The asterisk (*) indicates a statistically significant difference compared with WT ($p < 0.05$, unpaired t-test).

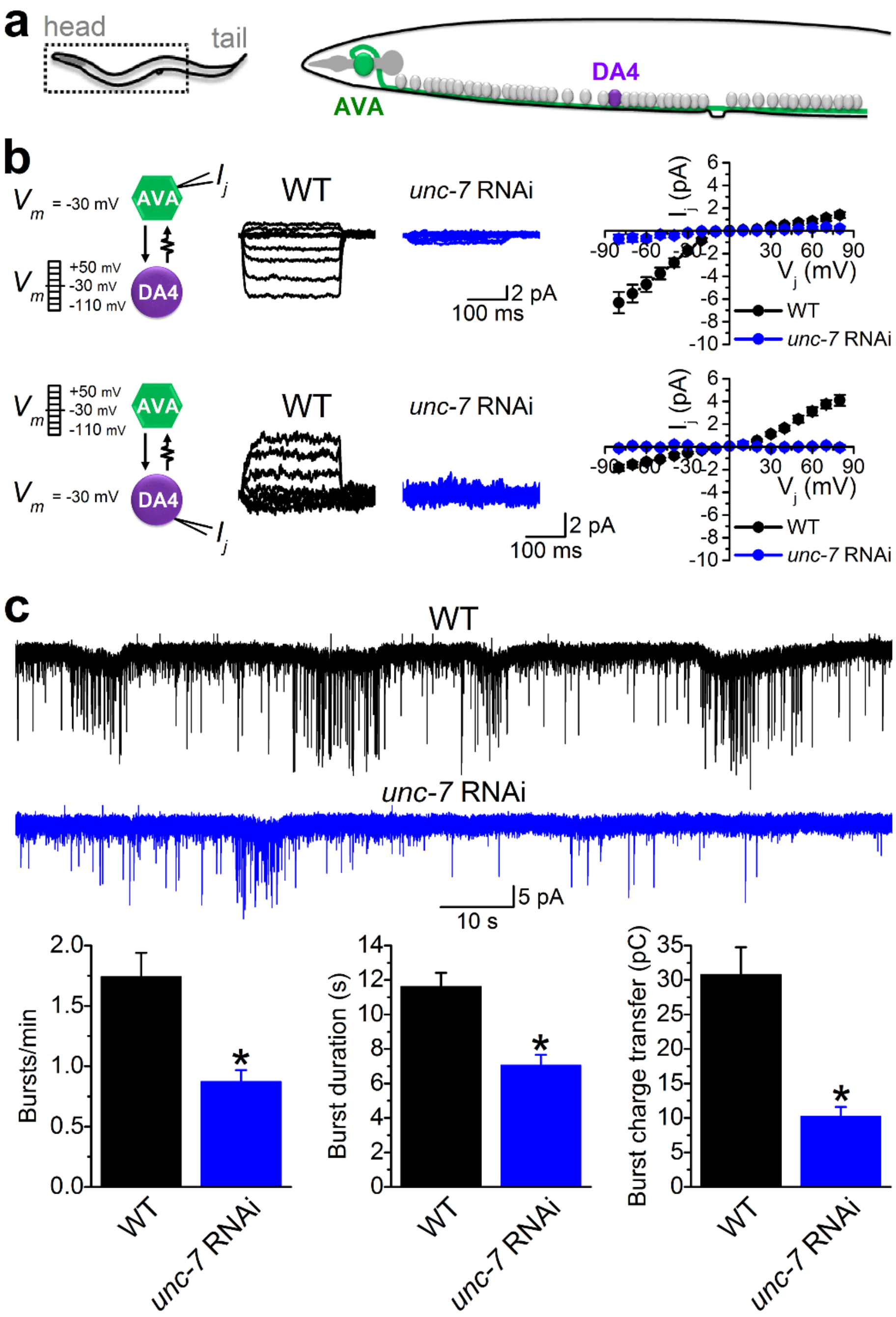

Supplementary Figure 6. Deficient electrical coupling inhibits chemical synaptic transmission between AVA and DA4. (**a**) Diagram showing the locations of AVA and DA4. (**b**) Knockdown of *unc-7* specifically in AVA (n = 7) abolishes the I_j observed in wild type (WT, n = 8). I_j was recorded from one neuron held constantly at −30 mV (neuron #1) while the other neuron (neuron #2) was stepped to various membrane voltages (V_m) from a holding voltage of −30 mV. $V_j = V_m$ of neuron #1 − V_m of neuron #2. Whole-cell membrane currents of the neuron receiving the voltage steps are not shown for clarity. (**c**) Knockdown of *unc-7* specifically in AVA (n = 9) causes great inhibition of PSC bursts in DA4 compared with WT (n = 8). Data are shown as mean ± SE. The asterisk (*) indicates a statistically significant difference compared with WT (p < 0.05, un-paired t-test).

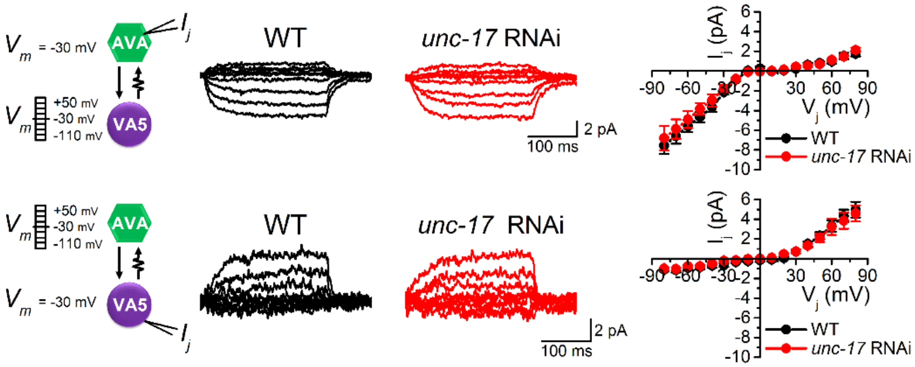

Supplementary Figure 7. Deficient chemical synaptic transmission does not alter electrical coupling between AVA and A-MNs. Junctional current (I_j) was recorded from either AVA or VA5 in wild type (WT, *n* = 12) and a worm strain in which *unc-17* is knocked down specifically in AVA interneurons (*n* = 7). I_j was recorded from one neuron held constantly at −30 mV (neuron #1) while the other neuron (neuron #2) was stepped to various membrane voltages (V_m) from a holding voltage of −30 mV. V_j = V_m of neuron #1 − V_m of neuron #2. Whole-cell membrane currents of the neuron receiving the voltage steps are not shown for clarity.

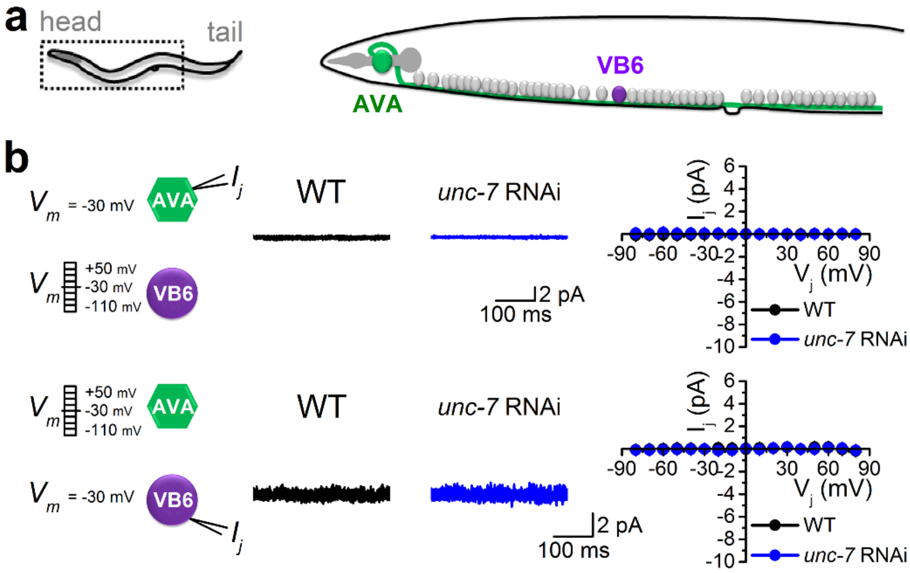

Supplementary Figure 8. VB6 is not electrically coupled to AVA in both wild type (WT) and AVA-specific *unc-7* knockdown strain. (**a**) Diagram showing the locations of AVA and VB6. (**b**) I_j was not detected between AVA and VB6 in either WT (*n* = 7) or the *unc-7* knockdown strain (*n* = 7). I_j was recorded from one neuron held constantly at −30 mV (neuron #1) while the other neuron (neuron #2) was stepped to various membrane voltages (V_m) from a holding voltage of −30 mV. V_j = V_m of neuron #1 − V_m of neuron #2. Whole-cell membrane currents of the neuron receiving the voltage steps are not shown for clarity.
